# An Integrated PCR Colony Hybridization Approach to Screen cDNA Libraries for Full-Length Coding Sequences

**DOI:** 10.1371/journal.pone.0024978

**Published:** 2011-09-15

**Authors:** Jacob Pollier, Miguel González-Guzmán, Wilson Ardiles-Diaz, Danny Geelen, Alain Goossens

**Affiliations:** 1 Department of Plant Systems Biology, Vlaams Instituut voor Biotechnologie (VIB), Gent, Belgium; 2 Department of Plant Biotechnology and Bioinformatics, Ghent University, Gent, Belgium; 3 Department of Plant Production, Faculty of Bioscience Engineering, Ghent University, Gent, Belgium; University of Münster, Germany

## Abstract

**Background:**

cDNA-Amplified Fragment Length Polymorphism (cDNA-AFLP) is a commonly used technique for genome-wide expression analysis that does not require prior sequence knowledge. Typically, quantitative expression data and sequence information are obtained for a large number of differentially expressed gene tags. However, most of the gene tags do not correspond to full-length (FL) coding sequences, which is a prerequisite for subsequent functional analysis.

**Methodology:**

A medium-throughput screening strategy, based on integration of polymerase chain reaction (PCR) and colony hybridization, was developed that allows in parallel screening of a cDNA library for FL clones corresponding to incomplete cDNAs. The method was applied to screen for the FL open reading frames of a selection of 163 cDNA-AFLP tags from three different medicinal plants, leading to the identification of 109 (67%) FL clones. Furthermore, the protocol allows for the use of multiple probes in a single hybridization event, thus significantly increasing the throughput when screening for rare transcripts.

**Conclusions:**

The presented strategy offers an efficient method for the conversion of incomplete expressed sequence tags (ESTs), such as cDNA-AFLP tags, to FL-coding sequences.

## Introduction

cDNA-AFLP is a widely used, robust, and reproducible tool for genome-wide expression analysis in any organism, without the need for prior sequence knowledge [1], [2]. The technique is derived from AFLP [3] and is based on the selective PCR amplification of restriction fragments from a double-stranded cDNA template. To this end, the double-stranded cDNA template is digested with restriction enzymes, followed by ligation of specific adapters to the sticky ends of the digested cDNA. Subsequently, a subset of the restriction fragments is amplified by PCR by using primers with a few selective nucleotides in addition to the sequence complementary to the adapter and restriction site sequences. The amplified cDNA fragments are visualized on high-resolution polyacrylamide gels, on which the intensity of the fragments reflects the relative abundance (copy number) of the corresponding genes across the samples [2], [4]. To identify the differentially expressed genes, the corresponding cDNA-AFLP tags are purified from the polyacrylamide gel, reamplified, and sequenced. However, the resulting cDNA-AFLP tag sequences most often do not correspond to FL-coding sequences and, thus, do not provide sufficient sequence information for functional characterization.

Obtaining a FL-coding sequence of a gene of interest from a non-model species without genome information might often prove to be a laborious and difficult task. One of the commonly used methods to obtain a FL cDNA clone starting from a partial clone such as a cDNA-AFLP tag or any other type of EST, is the rapid amplification of cDNA ends (RACE)-PCR strategy, in which adapters ligated to the 3′ and 5′ ends of the cDNA are used to selectively amplify these 3′ or 5′ cDNA fragments by PCR through a combination of gene-specific and adapter-specific primers [5]. A common problem associated with RACE-PCRs is the amplification of non-specific PCR products due to the presence of one of the primer sequences in all cDNAs [6]. Several methods have been developed to solve this problem, but most of them rely on extra enzymatic steps after completion of the first strand cDNA synthesis, which may introduce mistakes [7]. An alternative to the RACE-PCR strategy is the screening of cDNA libraries. To identify clones from a cDNA library that contain the gene of interest, labeled gene-specific DNA probes are hybridized to colonies or plaques transferred to a nylon membrane. This traditional manner of library screening is labour intensive, especially when screening for rare transcripts [8]. A more rapid approach to obtain a clone of interest from a cDNA library is by PCR screening of pooled clones. This approach requires arraying of individual clones into microtiter wells, and is therefore only practical for abundant transcripts [9]. Alternatively, Self-Ligation of Inverse PCR Products (SLIP) allows for the simultaneous screening for low abundant transcripts. In this technique, the plasmid of interest is amplified from a small aliquot of the cDNA library via inverse PCR, using primers designed in opposite orientation on the incomplete cDNA. The obtained PCR fragments are self-ligated, transformed into *Escherichia coli* and plated. Subsequently, individual colonies are picked for DNA isolation and sequencing [8], [10]. However, similar to various RACE-PCR approaches, this PCR-based library screening strategy is susceptible to amplification artefacts. Hence, because of the time-consuming and labor-intensive iterative screening of a number of cDNA libraries or the need for directed strategies to process individual clones in a RACE-PCR approach, to obtain the FL cDNA clone of large numbers of incomplete cDNAs in parallel, may prove to be a real challenge.

To overcome this bottleneck, we developed a medium-throughput cDNA library screening strategy that integrates PCR and colony hybridization. The fast and easy PCR methodology was combined with the robust colony hybridization technique, allowing the screening of approximately 100,000 cDNA clones for individual cDNA clones, in a rapid and straightforward manner. The screening strategy can be divided into three basic steps. First, 100,000 cDNA colony-forming units or clones were divided in 12 pools of approximately 8,000 cDNA clones each. The pools (inoculated from the primary transformants) were grown overnight in liquid medium to increase the cell mass and each culture was then split into two halves. In the second step, of one half of each of the overnight-grown cultures, the plasmid DNA was extracted. The extracted pool plasmid DNA was used as PCR template to identify the pool containing the cDNA clone corresponding to the incomplete cDNA tag. In step three, the remaining half of the overnight-grown culture was plated out on Petri dishes for classical colony hybridization, using the incomplete cDNA tag as probe. The developed screening strategy was used to screen cDNA libraries of three medicinal plants, *Maesa lanceolata* (false assegai), *Glycyrrhiza glabra* (liquorice), and *Panax ginseng* (ginseng) and allowed to pick up over 100 FL clones in two screening rounds, with a success rate of 67%.

## Results

### Identification of MlJAZ1

**Figure 1:**
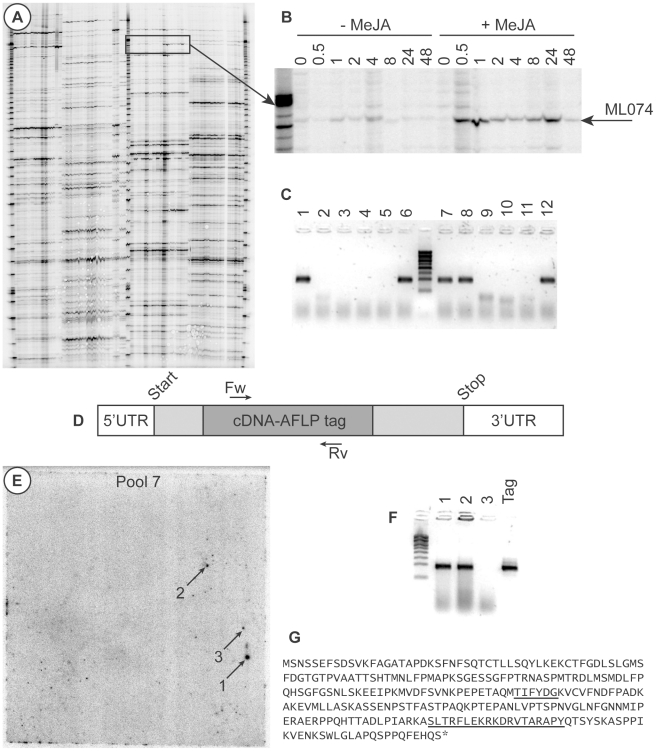
Identification of *MlJAZ1*. (A) cDNA-AFLP gel. (B) Detail of the cDNA-AFLP gel showing the MeJA response of the ML074 tag. (C) Result of the PCR screening, showing the presence of ML074 in pools 1, 6, 7, 8, and 12. (D) Schematic representation of *MlJAZ1*. (E) Autoradiogram of pool 7 after hybridization with the radioactively labeled ML074 tag; arrows mark the three candidate colonies. (F) Colony PCR on the candidate colonies. The cDNA-AFLP tag was used as template for the positive control. (G) Identification of the tify and Jas domains in the MlJAZ1 amino acid sequence.

In our research on secondary metabolism, we investigated the effect of methyl jasmonate (MeJA) elicitation on the transcriptome of medicinal plants by cDNA-AFLP transcript profiling. In one such study, the transcriptome of MeJA-elicited axenic shoot cultures of *M. lanceolata* was analyzed. A collection of MeJA-responsive gene tags was obtained, of which ML074 was strongly transcriptionally activated within 30 minutes after MeJA treatment (Figure 1A,B). The cDNA-AFLP tag was purified from the gel, reamplified as described [2], [11], and sequenced, revealing an EST of 449 nucleotides that was closely homologous to the jasmonate signaling repressor *JAZ1/TIFY10A* from *Arabidopsis thaliana*. To obtain the FL cDNA corresponding to the ML074 tag, a *M. lanceolata* Uncut Nanoquantity cDNA library (Invitrogen, Carlsbad, CA, USA) was screened with our approach. The primers for the PCR screening (forward and reverse, 5′-TTTATTCCCCCAGCACTCTG-3′ and 5′-TCGGAGCTTGCCTTACTAGC-3′, respectively) (Figure 1D) were developed on the cDNA-AFLP tag with the Primer3 program [12]. Subsequent screening of the pool plasmid DNA revealed that the clone was present in pools 1, 6, 7, 8, and 12 (Figure 1C). After colony hybridization on the membrane of pool 7, three candidate colonies could be observed on the resulting autoradiogram (Figure 1E). By means of colony PCR with the above-mentioned primers, two of the three candidate colonies were found to contain the clone of interest (Figure 1F). Sequencing of the two positive clones demonstrated that in both clones an identical FL open reading frame (ORF) of 819 nucleotides occurred, encoding a protein of 273 amino acids, hereafter referred to as MlJAZ1. The 5′ untranslated region (UTR) present in clone 1 was 153 nucleotides long, whereas the 5′ UTR of clone 2 was only 36 nucleotides long. The sequence of clone 1 was deposited in the GenBank with accession number JF313904. Analysis of the obtained MlJAZ1 sequence showed that the protein contained the characteristic tify and Jas domains of the JAZ protein family (Figure 1D,G). The tify domain and the C-terminal Jas domain are characterized by the highly conserved TIF[F/Y]XG and SLX_2_FX_2_KRX_2_RX_5_PY amino acid sequences, respectively [13]–[19].

### In-parallel screening for *Maesa lanceolata* genes

**Figure 2:**
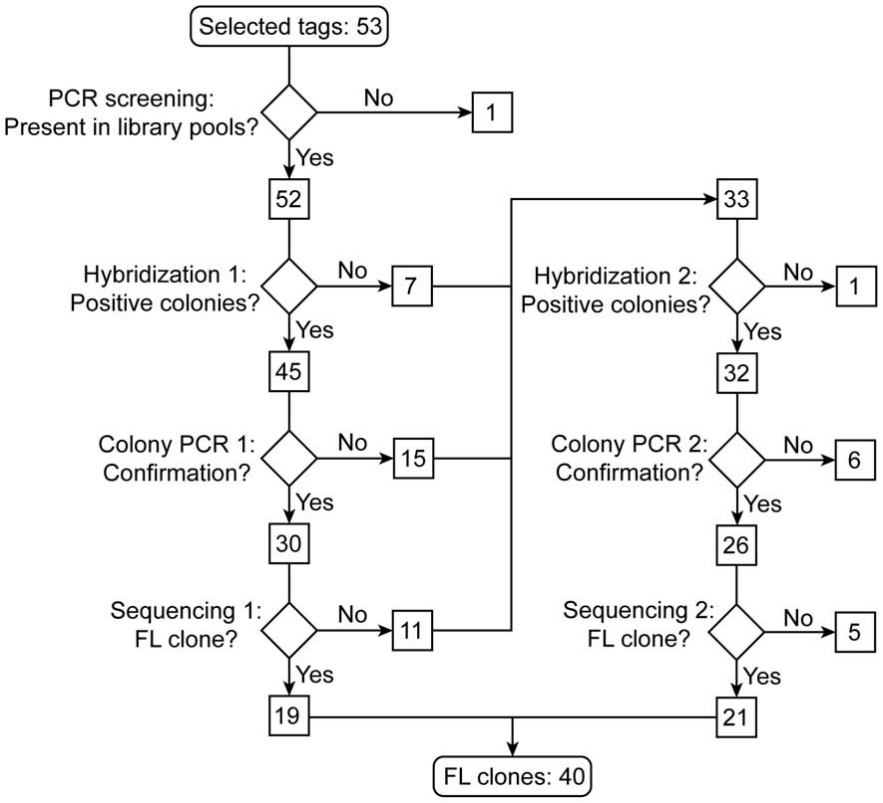
Flow-chart of the screening for FL clones of selected *M. lanceolata* tags. For details, see text.

Besides ML074, 52 other *M. lanceolata* cDNA-AFLP tags were selected for further analysis. PCR screening of the pool plasmid DNA indicated that clones corresponding to 52 of the 53 selected tags were present in at least one of the 12 library pools (Figure 2). Furthermore, clones corresponding to 23 tags were present in all the pools, suggesting a high representation in the library. Subsequently, a first round of colony hybridizations were performed, in which the 52 radioactively labeled cDNA-AFLP tags were hybridized on one membrane of a pool for which the presence of a clone corresponding to the cDNA-AFLP tag was confirmed by the PCR screening. For 45 tags, at least one candidate colony was identified, but colony PCR revealed that for 15 tags, all identified candidate colonies were false positives. Thus, for 30 cDNA-AFLP tags, confirmed colonies were obtained and subsequently sequenced, until a clone with a FL-coding sequence was identified, resulting in the identification of FL sequences for 19 of the 53 initially selected cDNA-AFLP tags (36%). For the remaining 33 cDNA-AFLP tags that occurred in one or more of the pools, and for which no corresponding FL-coding sequence was identified, a second round of hybridizations was performed (Figure 2), until for every cDNA-AFLP tag a candidate colony was identified, or until all the pools with a hit in the PCR screen were exhausted. For all, but one, of the 33 remaining tags, candidate colonies were identified. Subsequent colony PCR showed that for six tags, all identified colonies were again false positives and, thus, colony PCR confirmed clones were found for 45 of the 53 initially selected cDNA-AFLP tags. Sequencing of the candidate colonies allowed us to identify 21 more FL clones in the second hybridization round, ending with a total of 40 confirmed FL clones out of the 53 initially selected cDNA-AFLP tags (75%); colony PCR confirmed clones corresponding to the five remaining cDNA-AFLP tags all contained truncated versions of the corresponding ORF. A flow-chart of the screening for FL clones of the *M. lanceolata* cDNA-AFLP tags is given in Figure 2.

### Panax ginseng and Glycyrrhiza glabra cDNA library screening

Besides *M. lanceolata*, cDNA-AFLP tags were selected from two other medicinal plants treated with MeJA, *P. ginseng* and *G. glabra*. For *P. ginseng*, a total of 41 cDNA-AFLP tags were selected, of which the screening PCR confirmed the presence of 38 corresponding clones in the approximately 100,000 screened clones of the cDNA library. For *G. glabra*, a total of 69 tags were selected and the PCR screening confirmed the presence of 59 clones. FL genes of *G. glabra* and *P. ginseng* were screened for as for *M. lanceolata* and 47 and 22 FL genes were obtained out of the 69 (68%) and the 42 (54%) initially selected tags, respectively. In conclusion, by combining the three screenings, we identified a FL clone corresponding to 109 cDNA-AFLP tags out of a selection of 163 tags, reaching an overall success rate of 67%. The overview (Table 1) illustrates that the success rate is higher for enzyme-encoding genes than for transcription factors, possibly because the latter are rarer represented in the library than the former.

**Table 1:** Overview of the cloning efficiency.

Species	Enzymes	Transcription factors	Total
*M. lanceolata*	32/37 (86%)	8/16 (50%)	40/53 (75%)
*G. glabra*	36/49 (73%)	11/20 (55%)	47/69 (68%)
*P. ginseng*	22/41 (54%)	Not selected	22/41 (54%)

## Discussion

The designed “EST-to-FL conversion” strategy described here has proven efficient in the screening for FL clones corresponding to a large number of cDNA-AFLP tags. During the screening process, two hybridization rounds could be done per week, each including 12 membranes, for which the following schedule was applied. On day 1, probes were labeled, and hybridizations were performed overnight. On day 2, the membranes were washed thrice to remove excess probe and exposed to the autoradiography films for 2 days. On day 4, the autoradiography films were developed and the membranes were stripped prior to a second round of overnight hybridizations. On day 5, the membranes were washed and exposed to the autoradiography films for 2 days. On day 8, the second batch of films were developed and the membranes stripped, and another round of hybridizations could be started. This system allowed us to use the membranes for up to six hybridizations rounds without significant loss of hybridization potential.

Furthermore, with this hybridization system, multiple probes can be utilized in a single hybridization event. Therefore, 50 ng of each probe was mixed, prior to labeling, in a double volume of the probe labeling reaction mixture and so, up to six probes on a single membrane could be hybridized at once. Although the hybridization throughput is highly increased and, thus, less handling with radiolabeled substrates is necessary, the subsequent colony PCR step becomes more elaborate as a consequence, because every identified candidate colony has to be checked with all of the primer combinations corresponding to all of the probes used in the hybridization. Nonetheless, despite this drawback, the use of multiple probes proved to be beneficial in the second round of hybridizations (Figure 2), when transcripts without positive colonies in the first hybridization round were screened.

After hybridizations, candidate colonies were confirmed by colony-PCR and subsequently sequenced. For instance, for the 53 selected *M. lanceolata* tags, a total of 167 plasmids were sent for sequencing. Subsequent sequence analysis showed that 73/167 (43.7%) plasmids corresponded to FL clones, and at least one FL clone was obtained for 40 of the 53 selected tags. Although the applied strategy is not specific for FL clones, it allows medium-throughput isolation of candidate clones, provided the cDNA library is of sufficient quality. The latter may be one of the most important criteria for the success rate of FL-clone identification, not only for the approach we have developed here but principally for any FL screening methodology. For instance, the screening of a *Bupleurum falcatum* Uncut Nanoquantity cDNA library with a lower percentage of FL-clones (28.3%) pointed to a positive correlation between the quality of the cDNA library and the success rate of the FL cloning method. Of the 64 selected *B. falcatum* cDNA-AFLP tags, 59 were confirmed to be present in at least one of the library pools by PCR screening, which was similar to the number obtained with the *M. lanceolata* cDNA library. However, the subsequent first round of colony hybridizations allowed identifying 8 FL clones only (13.5% success rate), compared to for instance the 19/52 in the *M. lanceolata* library (36.5% success rate, Figure 2).

To increase the rate of FL clone identification, colony-PCR can be performed using a forward primer corresponding to the vector, at the 5′ end of the cDNA, and a reverse primer corresponding to the tag or EST, and subsequently, the clone that yields the largest PCR fragment can be subjected to sequencing. Alternatively, RACE-PCR-based approaches may have a higher chance of finding a FL clone for a particular tag, however, these methods might be more susceptible to PCR amplification artefacts and be more labour intensive, hence it might be troublesome to implement them to obtain the FL clone of large numbers of incomplete cDNAs in parallel.

Similar to our strategy, SLIP allows in parallel screening of a cDNA library for FL clones [8], [10]. Eliminating the use of radioactivity and the colony hybridization step, the SLIP strategy allows the recovery of FL clones from plasmid cDNA libraries in 5 days [8]. It should be noted though, that also in our strategy, the use of radioactivity can be eliminated by using non-radioactive techniques to perform colony hybridisations. Indeed, the approach that we have developed is compatible with any method of probe labelling. Hence it provides a valid alternative strategy to SLIP that can be implemented in every lab to screen a cDNA library for FL clones in a medium-throughput manner.

Here, the usefulness of our “EST-to-FL conversion” strategy has been shown for one class of incomplete cDNAs, i.e. cDNA-AFLP tags, but, principally, it can be applied to any type of incomplete tag sequence. Indeed, several commonly used techniques generate often very large collections of incomplete cDNAs or ESTs. For instance, in the latest GenBank database of EST release (dbEST release 060111, http://www.ncbi.nlm.nih.gov/dbEST/index.html), a total of 69.7 million ESTs were released. Similarly to cDNA-AFLP tags, these ESTs are typically between 200 and 500 nucleotides long. Contigs can be generated from large collections of ESTs from a single organism and allow the identification of the FL ORFs of the genes of interest. However, for most of the organisms, the EST collection is too small to generate such contigs. The presented strategy might be applied to pick up the FL ORFs corresponding to the published ESTs. Primers designed on the released EST sequences can be used for the PCR screening and the resulting PCR product can be purified and labeled to use it as a probe to pick up the full clone via the colony hybridization step of the presented strategy.

## Materials and Methods

### Pool preparation

As starting material, Uncut Nanoquantity cDNA libraries (custom-made by Invitrogen, Carlsbad, CA, USA) were used. Approximately 120,000 clones of the original library were transferred to a 2-mL tube and Lysogeny Broth (LB) medium containing 50 µg/mL kanamycin (LBKAN50) was added to a total volume of 1.5 mL. This library dilution was used to inoculate 12 flasks, each containing 10 mL of LBKAN50 medium with approximately 8,000 cDNA clones each (100 µL of the prepared library dilution per flask). The resulting *E. coli* cultures were incubated at 37°C with shaking at 300 rpm, for only 12–16 hours to limit out-competition, before pool plating.

### PCR screening

For the plasmid DNA extraction, 5 mL of each 10-mL culture was used. To identify the pools that contained the cDNA clone corresponding to the target cDNA-AFLP tag, 50 ng of the pool plasmid DNA was taken as PCR template. Specific PCR primers were designed based on the cDNA-AFLP tag sequences with the Primer3 program [12].

### Pool plating

The titer of the remaining 5 mL of each 10-mL culture was estimated by plating a serial dilution of the cultures on LBKAN50 plates, followed by overnight incubation at 37°C. Subsequently, of each pool, approximately 10,000 cDNA clones were plated on LBKAN50 medium containing 15 g/L agar in 24×24 cm Petri dishes and grown overnight at 37°C. Prior to colony lifting, the plates were pre-cooled at 4°C.

### Colony lifting

The colonies grown on the 24×24 cm Petri dishes were lifted on Hybond-N^+^ membranes (GE-Healthcare, Little Chalfont, UK), according to the manufacturer's instructions. The DNA was cross-linked to the membranes by UV irradiation.

### Probe labeling

Probes were re-amplified by PCR from the cDNA-AFLP tag [2] and subsequently labeled with ^32^P via a random priming reaction by incubating 25 µL of the following mixture at 37°C for 6 hours: 50 ng denatured probe, 1× bovine serum albumin (0.1 mg/mL), 5 mM MgCl_2_, 0.5 mM dithiothreitol (DTT), 20 mM 2-(*N*-morpholino)ethanesulfonic acid (MES), 20 µM of dATP, dGTP, and dTTP each, 12.5 µCi deoxycytidine α-^32^P (500 µCi/mmol dCTP Easy Tide) (Perkin Elmer, Waltham, MA, USA), 1 ng hexanucleotide random primer (Roche Applied Science, Brussels, Belgium), and 5 U Klenow Fragment of DNA Polymerase I (GE-Healthcare). The labeled probes were stored at −20°C until used for hybridization.

### Hybridization

The membranes were pre-wetted for 30 min at 65°C in preheated hybridization buffer (0.4 M Na_2_HPO_4_, pH 7.2, 1 mM ethylenediaminetetraacetic acid (EDTA), 7% (w/v) sodium dodecyl sulfate (SDS), after which the denatured radioactive probe was added. After overnight hybridization at 65°C, the membranes were washed thrice with preheated washing buffer (0.04 M Na_2_HPO_4_, pH 7.2, 1 mM EDTA, and 5% (w/v) SDS) at 65°C. Subsequently, excess buffer was drained off. The membranes were packed in SaranWrap and exposed to Kodak™ BioMax films (Perkin Elmer) for 2 days. The membranes can be re-used for hybridization after stripping by washing them thrice with preheated stripping solution (1 mM EDTA and 0.1% [w/v] SDS) at 75°C.

### Clone confirmation

Candidate colonies were picked from the 24×24 cm Petri dishes and checked by colony PCR with the primers designed on the cDNA-AFLP tag. Positive clones were sequenced with the primers designed on the pENTR222 vector of the Uncut Nanoquantity cDNA library, 5′-ACGACGGCCAGTCTTAAGCTCGG-3′ and 5′-ACCATGTAATACGACTCACTATAGG-3′.
